# Poisoning by Strychnine

**Published:** 1857-06

**Authors:** J. Ward Davidson

**Affiliations:** Nevada, Iowa


### POISONING, BY STRYCHNIA?

BY J. WARD DAVIDSON, M. D.

A somewhat singular case of supposed poisoning by Strychnia, occurred in this vicinity a short time since.

Mr. D—— residing some twelve miles northwest of this place, procured strychnia for the purpose of destroying wolves. He placed the prepared bait on the 31st December, and on the following morning with a neighbor went out to examine if anything had been accomplished. A short distance from where the bait had been left, they discovered a dead wolf which had evidently, previous to death, disgorged the poisonous food. This had apparently been again eaten by another wolf, and following its tracks in the snow they soon came upon the animal lying dead.

Mr. D—— threw this last over his shoulder holding it by its forelegs with its head near his face, and thus bore it to its house.

Shortly after his arrival home, Mr. D., was seized with all the symptoms (in a mild form,) of poisoning by strychnia. He at once drank nearly two pints of melted lard, which relieved the symptoms for the time.

On the 10th of January he attempted to prepare another bait, but was seized before completing its preparation with the same symptoms, which he treated in the same way, with like results as before.

Again, on the 19th of January, he was attacked in the same manner, when my partner, Dr. Adamson, was called in. The patient was greatly prostrated by the violent spasms he had suffered, but these were becoming gradually milder, and were soon controlled. On the 28th of same month, I learn, he had a slight recurrence of the symptoms.

Now, if the strychnia was the cause of the spasmodic difficulty in the above case, it may be considered one of rare interest. The manner of receiving the poisonous influence, and the recurrence of the symptoms at two stated periods after its use, &c., &c., would be out of the ordinary course. Hahnemann's "medicinal aura" or rather the excited imagination of the patient may perhaps be considered as the first cause, and perhaps the only cause of the disagreeable symptoms. In either case, it was both strange and interesting to us.

Was it a case of idiopathic tetanus? Certainly it had no traumatic origin.

In conclusion, let me remark, that the carcasses of the wolves poisoned did not freeze, although exposed for several days to extreme cold—several degrees below zero. This I thought somewhat curious.

NEVADA, Iowa, February 3d, 1857.

